# High Catalytic Activity of Co_x_Pt_100−x_ Alloys for Phenolic Compound Reduction

**DOI:** 10.3390/nano14070599

**Published:** 2024-03-28

**Authors:** Oana-Georgiana Dragos-Pinzaru, Gabriela Buema, Luiza Racila, Gabriel Ababei, Firuta Borza, George Stoian, Ibro Tabakovic, Nicoleta Lupu

**Affiliations:** 1 National Institute of R&D for Technical Physics, 700050 Iasi, Romania; odragos@phys-iasi.ro (O.-G.D.-P.); gababei@phys-iasi.ro (G.A.); fborza@phys-iasi.ro (F.B.); gstoian@phys-iasi.ro (G.S.); nicole@phys-iasi.ro (N.L.); 2 Department of Electrical and Computer Engineering, University of Minnesota, Minneapolis, MN 55435, USA; tabakovicibro@gmail.com

**Keywords:** Co_x_Pt_100−x_ alloys, catalytic activity, 4-nitrophenol reduction, water treatment

## Abstract

In this study, we report the influence of the Pt concentration in Co_x_Pt_100−x_ alloys on the catalytic activity of the alloys for 4-nitrophenol (4-NP) reduction. More precisely, a series of Co_x_Pt_100−x_ alloys with a Pt concentration ranging between 60% and 95% were prepared using electrodeposition at controlled potentials from stable hexachloroplatinate aqueous solution. The Pt concentration was tuned by varying the electrodeposition potential from −0.6 to −0.9 V. The changes in the Co_x_Pt_100−x_ alloy microstructure and crystalline structure have been investigated using SEM and TEM analysis. Our results show that the microstructure and the crystalline structure of the as-prepared materials do not depend on the electrodeposition potential. However, the catalytic activity of Co_x_Pt_100−x_ alloys is closely correlated with the potential applied during electrochemical synthesis, hence the Pt content. We demonstrated that the synthesized materials present a high catalytic activity (approx. 90%) after six cycles of reusability despite the fact that the Pt content of the as-prepared alloys decreases. The easy preparation method that guarantees more than 97% catalytic activity of the Co_x_Pt_100−x_ alloys, the easy recovery from solution, and the possibility of reusing the Co_x_Pt_100−x_ alloys are the benefits of the present study.

## 1. Introduction

Water pollution (defined as alteration in the physical, chemical and biological properties) is an increasingly dangerous problem, representing currently one of the most important environmental risks to human health, affecting people across the world. Numerous deadly diseases have a close relationship with the ingestion of contaminated water. Water issues are perceived as one of the major environmental concerns at national and international levels. To deal with the plethora of substances that need to be removed from wastewater in order to protect humans, animals, and the environement from exposure to pollutants, modern strategies have to be developed to remediate wastewaters and to limit the polution damages. Moreover, against the background of the increasing global demand for water, climate change and the expansion of arid and semi-arid regions, the requirements for water treatment will increase considerably in the future. In this context, the development of new devices, which can be used for the treatment of wastewaters, is imperative and represents an important socio-economic and environmental issue.

The heavy metals, anionic/cationic dyes, phenolic compounds, per- and polyfluoroalkyl substances, drugs, oils, and microplastics from various industry sectors can negatively affect water quality even in low concentrations and cause serious problems.

The phenolic compound, namely, 4-nitrophenol (4-NP), is recognized as a hazardous pollutant with a negative impact on human health [1,2,3,4]. For this reason, it is suggested that 4-NP concentrations in water must be kept below 10 ppb [4]. Different methods were applied for the treatment of wastewaters contaminated with this type of high-risk contaminants [5,6,7], the catalytic reduction of 4-NP to 4-aminophenol (4-AP) with NaBH_4_ being the most commonly used technique [8,9,10]. It was reported that the 4-AP compound is a lesser dangerous contaminant [11,12,13].

It was noted by researchers that different materials have the ability to treat 4-NP-contaminated wastewaters, including nanosheets CoMn_2_O_4_ [14], Ni_2_P/biocarbon composite [15], MOF (Metal–Organic Frameworks)-derived CuFe@C catalysts [16], NiFe_2_O_4_@TiO_2_@PDA-Ag nanocatalyst [17], Fe_2_O_3_-NiO-embedded calcium alginate-carboxymethyl cellulose composite [18], silver-decorated magnetic Fe_3_O_4_/alginate polymeric surfactant [19], amino-rich Cu(I)–I coordination polymer [20], concave gold nano-arrows [21], biogenic silver nanoparticles [22], bismuth/reduced graphene oxide nanocomposites [23], porous nitrogen-doped reduced graphene oxide [24], and so on.

Among the materials involved in 4-NP reduction, recently, our research group reported, for the first time, results concerning the catalytic performance of the Co_x_Pt_100−x_ alloys in the shape of thin films for the 4-NP to 4-AP conversion [25]. Taking into consideration the promising results of the previous research and the fact that, to the best of our knowledge, there are not many in-depth investigations of the catalytic activity of Co_x_Pt_100−x_ alloys for 4-NP reduction reported in the literature, the main idea of the present study is focused on the synthesis of a new series of Co_x_Pt_100−x_ alloys prepared using electrodeposition at different controlled potentials (−0.6 V, −0.7 V, −0.8 V, and −0.9 V) from stable hexachloroplatinate aqueous solution for 4-NP compound reduction by investigating different working parameters. The preparation method is advantageous because no expensive equipment (such as sputtering equipment or furnaces) is necessary for the catalyst synthesis. The electrochemical synthesis root is a cheap and easy to employ preparation method, which allows for good control of the electrodeposited material characteristics. The replacement of Pt by other metals (especially non-noble metals) with comparable catalytic performances is a major challenge for the research community. The synthesis of new bimetallic catalysts by partially replacing the noble metal with a non-noble one (e.g., replacement of Pt with Co), can be a reliable solution to solve the noble metal shortage.

Another objective was focused on the characterization of synthesized Co_x_Pt_100−x_ alloys using SEM, XRD and TEM analysis. The evolution of the Pt content in the Co_x_Pt_100−x_ alloy in function of the plating potentials is also presented. The reusability study for multiple cycles was conducted to confirm the applicability of Co_x_Pt_100−x_ alloys for the catalytic reduction of 4-NP to 4-AP. A comparison of the data obtained in the present study with data obtained by others on different materials involved for 4-NP reduction will be also presented.

The characterization of Co_x_Pt_100−x_ alloys, after the reusability investigation, was performed using SEM analysis, which demonstrates no changes in the structure of materials.

The results of the current study demonstrate that the developed Co_x_Pt_100−x_ alloys can be applied for the catalytic reduction of 4-NP to 4-AP. The reusability study showed good stability for the Co_x_P_t100−x_ alloys, and the catalytic activity was approx. 90% after six cycles.

The use of Co_x_Pt_100−x_ alloys represents a successful approach for solving environmental problems created by this type of phenolic contaminant.

## 2. Materials and Methods

### 2.1. Chemicals

The reagents used for the synthesis of materials were supplied by Alfa Aesar: H_2_PtCl_6_ (99.9% purity), CoSO_4_*7H_2_O (98% purity), H_3_BO_3_ (99.8% purity), NH_4_Cl (99.5% purity), and sodium saccharine (99% purity).

For the catalytic investigation, the 4-NP reagent (99% purity) was purchased from Thermo Scientific (Waltham, MA, USA), and the Sodium borohydride (NaHB_4_) was purchased from Alfa Aesar (98% purity). The reagents were used as received.

### 2.2. Synthesis of Materials

The Co_x_Pt_100−x_ alloys’ thin films (x = 5, 10, 28, and 40) have been prepared using electrodeposition from a stable hexachloroplatinate solution, containing 0.00386 M H_2_PtCl_6_, 0.1 M CoSO_4_*7H_2_O, 0.4 M H_3_BO_3_ and 0.3 M NH_4_Cl. As additive, we used sodium saccharine 0.00389M. The electrodeposition process parameters (such as applied potential, time on and time off), as well as the electrochemical bath characteristics (such as composition, pH value and additives), have been selected based on previously obtained results [25,26], where we found that the saccharin addition into the electrochemical bath improves the thin film layer mechanical properties (the saccharin is a well-known additive used for smoothening and stress relieving electrodeposited thin films). We also found that the saccharine favors the preparation of the CoPt alloys with a hcp crystalline structure. Another important parameter that influences the crystalline structure of the as-deposited CoPt alloys is the electrochemical bath pH value. Our previous results clearly show that the increase in the pH value leads to the preparation of CoPt alloys with an hcp structure [25]. As a consequence, the electrodeposition was carried out at pH 5.5, adjusted by using 0.1 M NaOH aqueous solution. As substrate, a 50 nm Au sputtered seed layer with a 5 nm Ta adhesion layer on oxidized Si wafers was used. The controlled potential electrodeposition was performed by exposing 1 cm^2^ of Au sputtered thin layer to the Co_x_Pt_100−x_ electrodeposition solution. Prior to the electrodeposition, nitrogen was purged into the electrochemical bath for 25 min. Since the electrodeposited alloy composition strongly depends on the potential values applied during the synthesis, the electrodeposition potential was varied from −0.6 to −0.9 V and was applied for a time on of 2.5 s. Moreover, during the Co_x_Pt_100x_ alloy electrodeposition, a rest potential of −0.1 V was applied for 1 s.

Table 1 summarizes the synthesis conditions for the preparation of all materials.

### 2.3. Characterization of Materials

The as-prepared Co_x_Pt_100−x_ thin film average thicknesses were measured by using an Alpha Step IQ stylus-based surface profiler (KLA-Tencor, Milpitas, CA, USA). The plating rate, expressed as a ration thickness (nm) versus time (s), was calculated for all the materials employed in this study. The materials’ surfaces were investigated using Scanning Electron Microscopy (SEM) using a NEON40EsB CrossBeam System from Carl Zeiss GmbH (Aalen, Germany) and Transmissions Electron Microscopy (TEM) using an Ultra-High-Resolution Transmission Electron Microscope UHR-TEM LIBRA^®^200MC (Carl Zeiss GmbH, Aalen, Germany). Both electron microscopes were equipped with an Energy Dispersive X-ray module (EDX), which was used for the determination of the materials’ composition.

The crystalline structure of the materials employed in this study was analyzed using Selected Area Electron Diffraction (SAED) with the TEM UHR-TEM LIBRA^®^200MC.

The crystalline structure of the electrodeposited materials was examined using X-ray diffraction (XRD) with a Bruker (Billerica, MA, USA) AXS D8-Advance X-Ray Diffractometer.

### 2.4. Catalytic Activity Experiments for 4-NP

The catalytic activity of the P1–P4 materials was established using the previously proposed method [26]. For the catalytic experiments, a volume of 2.5 mL of distilled water was put into contact with P1–P4 materials and further mixed with 40 μL of 4-NP (5 mM and 10 mM concentrations). The reaction was performed in the presence of 0.2 M NaHB_4_ (0.5 mL), which was freshly prepared. The samples were collected at different time intervals. The experiments were conducted under intermittent stirring at room temperature.

For the reusability investigation, the same conditions as in the case of catalytic experiments were applied for the optimum contact time of 20 min. After the time of 20 min was reached, P1, P2, and P3 materials were recovered from solution, washed with distilled water and immediately added in a new fresh solution of 4-NP.

The UV–Vis absorption spectra were recorded with Synergy HTX multimode reader (BioTek Instruments, Santa Clara, CA, USA). The peak intensity of collected samples was compared with the peak intensity of blank solution of 5 and 10 mM at 400 nm wavelength. It was demonstrated that 4-NP shows an absorption peak at 317 nm in the absence of NaBH_4_ solution, while after the addition of the NaBH_4_ solution, the absorption peak moves to 400 nm. This may be attributed to the formation of 4-nitrophenolate ion [27,28,29,30].

## 3. Results

### 3.1. Co_x_Pt_100−x_ Thin Layer Microstructure, Composition, Crystalline Structure and Thickness

**Figure 1:**
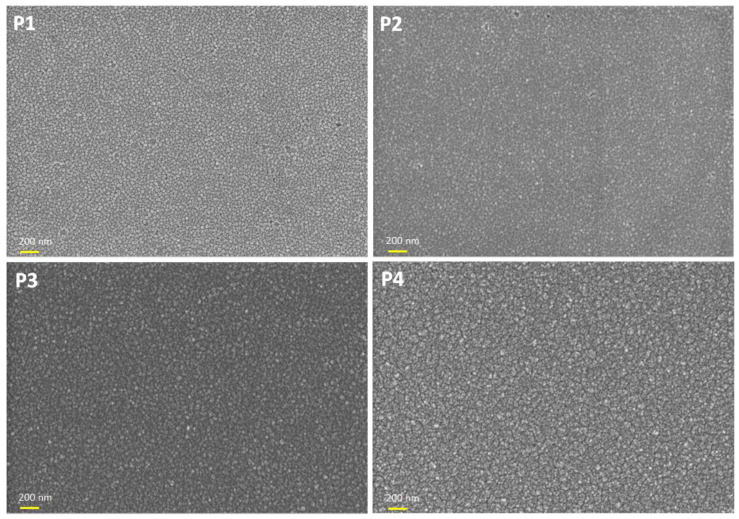
SEM images of the Co_x_Pt_100−x_ alloy surfaces prepared at different plating potentials.

The Co_x_Pt_100−x_ thin layer surface in function of the plating potential was analyzed using SEM, the result being presented in Figure 1. As shown in Figure 1, the SEM pictures do not highlight changes in the microstructure of the thin layers as a function of the applied potential during the electrodeposition process.

**Figure 2:**
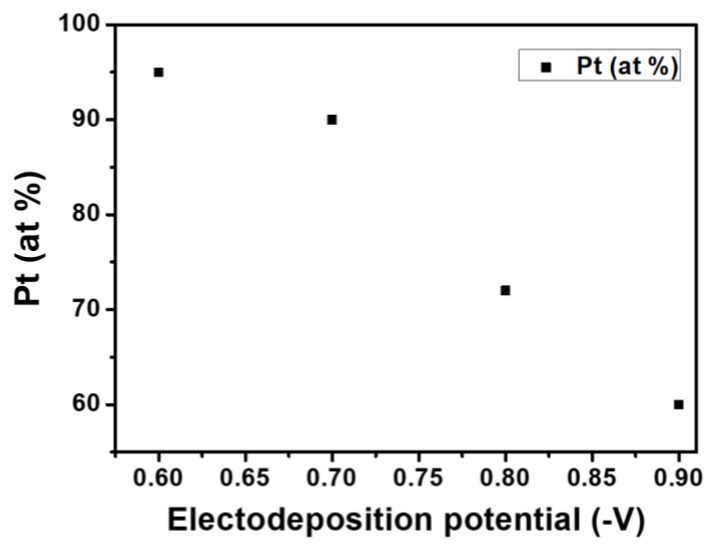
Evolution of the Pt content in the Co_x_Pt_100−x_ alloy in function of the plating potentials.

The composition of the as-prepared thin films was determined by the EDX analysis. The results, presented in Figure 2, show that the Co content of the alloy increases by increasing the absolute value of the applied potential during the electrodeposition.

**Figure 3:**
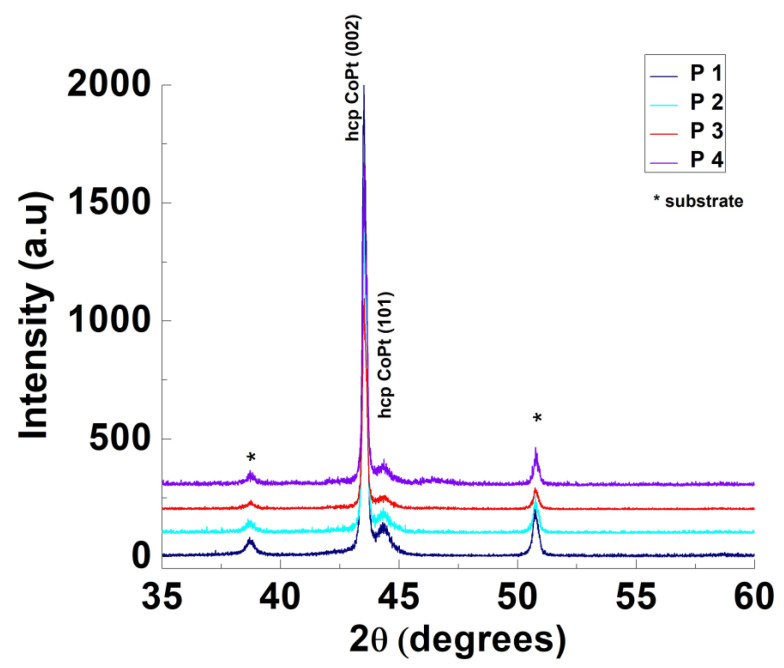
XRD patterns of CoPt thin films prepared by applying different electrodeposition potentials.

The XRD diffraction patterns (presented in Figure 3) confirm that all four materials (P1–P4) have a hexagonal hcp crystalline structure. This data are in good agreement with those presented in Ref. [26]. As can be observed from Figure 3, all the as-deposited materials present diffractions peaks at 2Θ = 43.5 and 44.5. These peaks can be assigned to (002) and (101) hcp structures, as found by Cortes and co-workers [31]. As can be observed from the XRD diffractogram presented in Figure 3, no impurity peaks are presented, indicating that the as-prepared materials are free of impurities.

**Figure 4:**
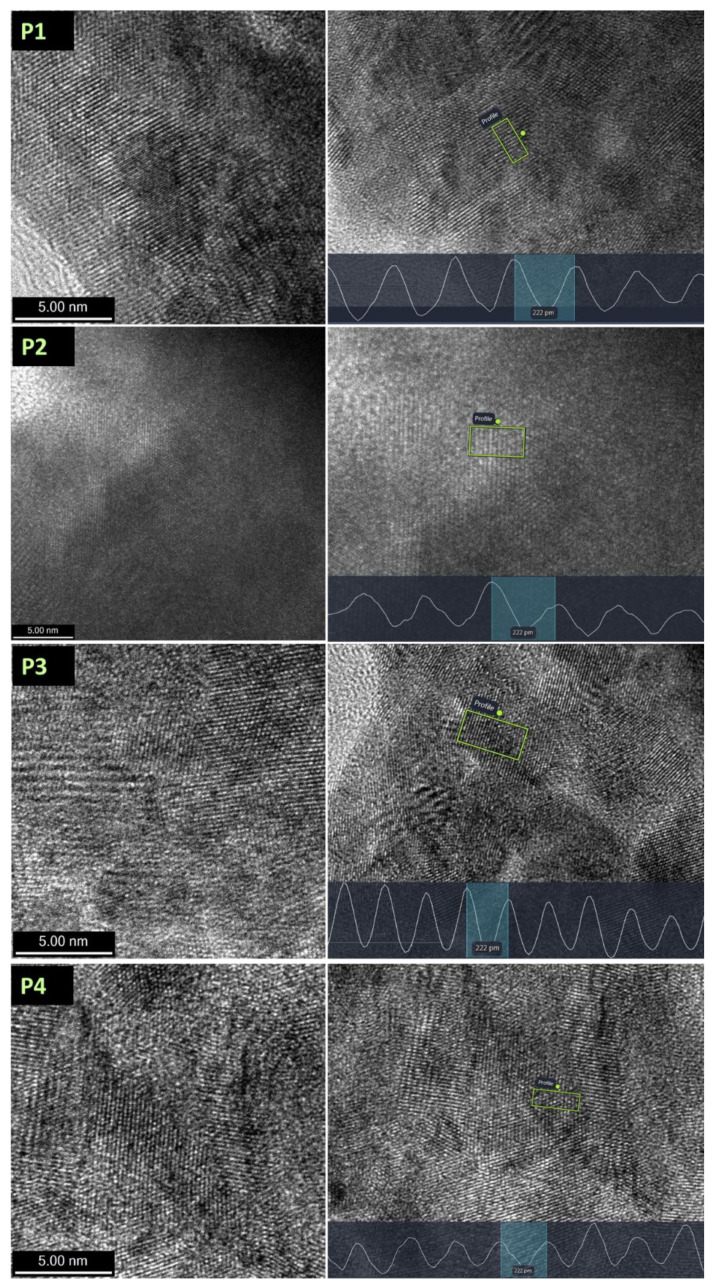
TEM image and d-spacing measurements of Co_x_Pt_100−x_ alloys prepared at different values of the applied potential.

The processed high-resolution TEM images’ lattice fringes show that all the Co_x_Pt_100−x_ alloys are crystalline. The analysis of the high-resolution TEM images shows that the lattice d-spacing of crystallites is 2.22 Å matching, in accordance with the Crystalography Open Database, cif file 1524154, the CoPt with a hexagonal structure with d-spacing of the (110) plane. The d-spacing measurements together with the TEM images are presented in Figure 4.

The measurements of the d-spacing show that the d = 2.22 Å, as can be observed from Figure 4. This value of the d-spacing corresponds to the hcp structure, showing that the P1–P4 materials present an hcp crystalline structure.

### 3.2. Catalytic Activity of P1–P4 Materials for 4-NP Reduction

**Figure 5:**
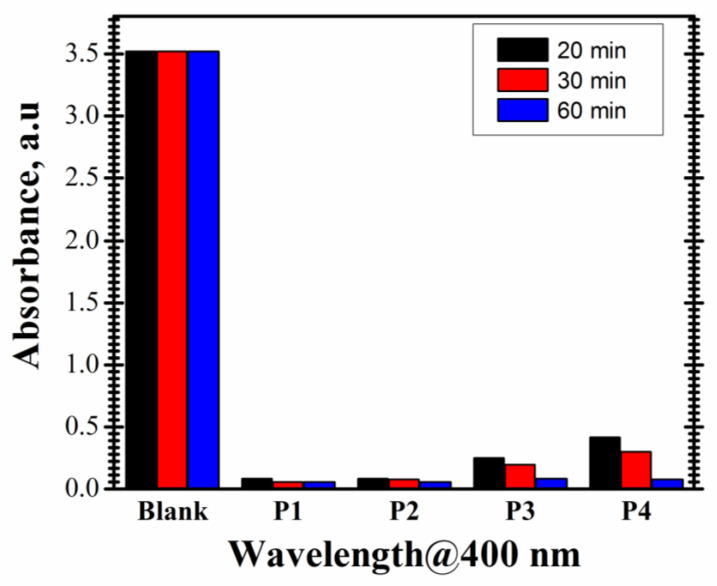
Catalytic activity of materials (4-NP concentration of 10 mM for 20, 30 and 60 min contact time, pH natural, room temperature, intermittent stirring).

A preliminary study was performed in order to establish the materials with the best catalytic activity for a 4-NP concentration of 10 mM and different values of the contact time. So, all materials were analyzed for 20, 30 and 60 min contact time using a 4-NP solution of 10 mM. The catalytic activity of P1–P4 materials is presented, by comparison, in Figure 5.

The results of the preliminary laboratory tests (from an optical point of view) showed that, in the first 10 min of contact time, no visible change in the solution color could be noticed (for this reason, no sample was collected for analysis). By increasing the contact time for more than 20 min, the color of each analyzed sample started to change. Thus, the results reflect the ability of materials to reduce the 4-NP to 4-AP.

The catalytic activity of prepared materials is dependent on the type of material. As shown in Figure 5, for the proposed working parameters, it can be highlighted that:

- The results obtained after 20 min of contact time indicate the same value of the catalytic activity of P1 and P2 materials, 97.61%, while P3 material presents a lower catalytic activity of 92.96%. The catalytic activity presented by P4 material is 88.23%.
- After 30 min, P1 and P2 present a similar catalytic activity to convert 4-NP to 4-AP (98.38% for P1 and 97.81% for P2), while P3 and P4 materials show a slightly different catalytic activity of 94.47% and 91.63%, respectively.
- After 60 min, the catalytic activity of P1 material remains unchanged (98.38%). P2 material shows a slightly higher catalytic activity of 98.35%, while P3 material shows a better catalytic activity of 97.56%. P4 material exhibits a visibly improved catalytic activity after 60 min of contact time compared to 30 min contact time (97.87% vs. 91.63%).
- When comparing the results at different time intervals, it can be observed that a shorter contact time is not favorable for the 4-NP reduction, especially for P4 material. For this synthesized material, the catalytic activity varies considerably with time.

The different catalytic behavior of the P1–P4 materials resides in the samples Pt concentration. Thus, P1 and P2 materials manifest similar catalytic properties for very small compositional differences (see Table 1). Starting with P3 and P4 materials, the Pt concentration decreases with 18% in the case of P3 material and with 30% for the material P4, respectively, leading to a decrease in the catalytic ability to convert 4-NP to 4-AP with 4% for P3 and with 7% for P4, respectively.

Taking into account that P1, P2, and P3 materials show good catalytic activity for a short period of contact time (30 min), they were selected for further investigation, including the study of other concentrations of 4-NP (5 mM) and of the reusability study.

#### 3.2.1. Effect of 4-NP Concentration

A parameter that can have an impact on the catalytic ability of the materials for 4-NP reduction is the concentration of 4-NP. In order to determine the effect of the 4-NP concentration on the catalytic activity of P1-P3 materials, the study was also performed for a lower concentration of the 4-NP solution, of 5 mM. The samples were collected after 10 and 20 min of contact time.

**Figure 6:**
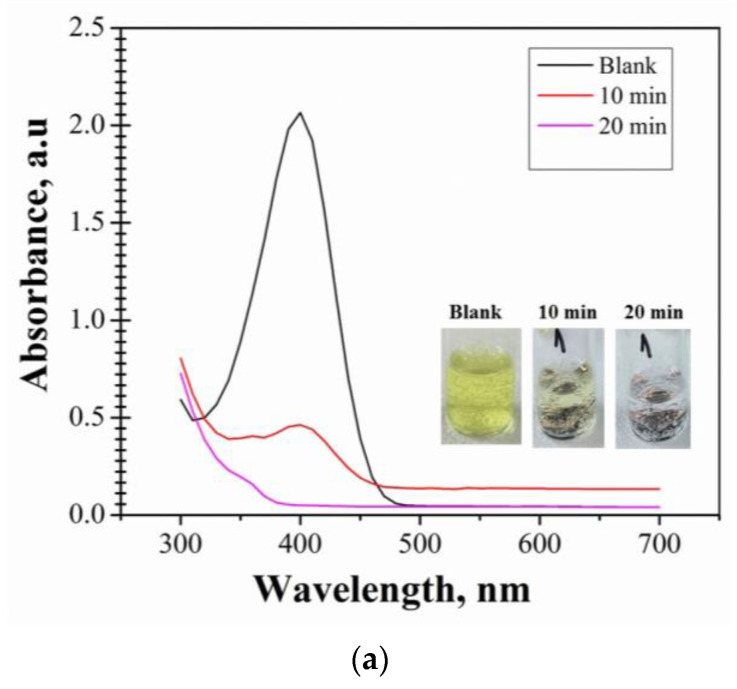

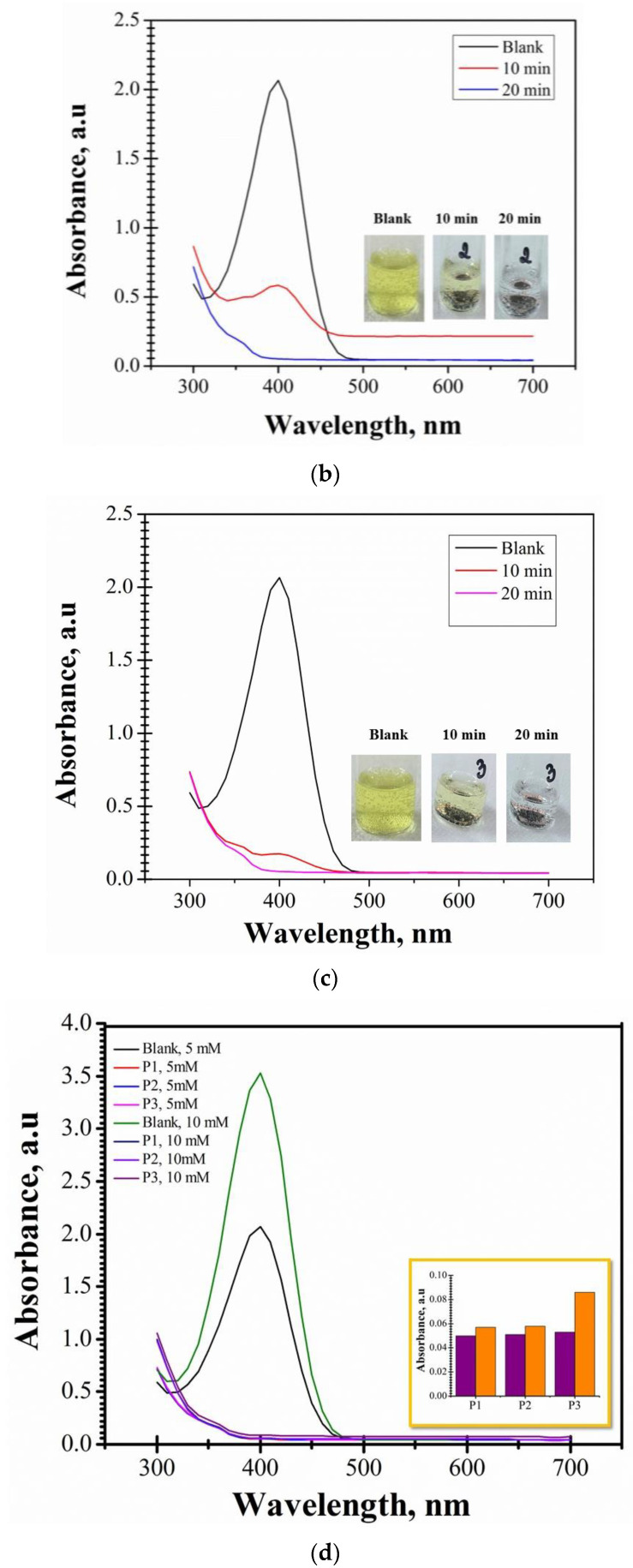
(**a**) Catalytic activity of P1 material at 4-NP concentration of 5 mM. (**b**) Catalytic activity of P2 material at 4-NP concentration of 5 mM. (**c**) Catalytic activity of P3 material at 4-NP concentration of 5 mM. (**d**) Catalytic activity of P1, P2, and P3 synthesized materials. Comparison of absorbance at a value of the wavelength of 400 nm: 4-NP concentration of 5 mM (purple) and 4-NP of 10 mM concentration (orange)—inset picture.

The data obtained at the concentration of 5 mM are presented in Figure 6a for P1 material, Figure 6b for P2 material, and Figure 6c for s P3 material.

The results demonstrate that the catalytic activity process for the P1–P3 materials is very fast. The peak intensity of collected samples visibly decreases after 10 min, as compared to the peak intensity of the blank solution. After 10 min of contact time, the materials show catalytic activity levels of 77.64%, 71.73%, and 91.58% for P1, P2, and P3, respectively. After 20 min in contact, the catalytic activity increases to 97.58%, 97.53%, and 97.43% for P1, P2, and P3 materials, respectively. From the inset pictures, a change in color of the solutions after a contact time of 10 min, compared to the blank solution, is noticed. After 20 min of contact time, the solution became colorless. Consequently, only about 20 min are needed to complete the reduction of 4-NP to 4-AP.

By comparing the results, P3 material presents a higher level of catalytic activity after 10 min (>90%) compared to the P1 and P2 materials, at a concentration of 5 mM.

The data obtained for 5 mM and 10 mM 4-NP concentration in the range of 300–700 nm are presented in Figure 6d, for comparison.

Comparison of the results of the effect of 4-NP concentration on the catalytic activity has led to the following observations:

- The P1 material presents a maximum catalytic activity of 98.38% after 30 min of contact time, at 10 mM concentration, while at 5 mM concentration the complete reduction of 4NP to 4-AP occurs after a shorter period of 20 min of contact time (97.58%);
- A higher catalytic activity of 98.35% and 97.56% was obtained for P2 and P3 materials at 10 mM concentration, but after a longer contact time of 60 min. The reduction of 4-NP to 4-AP after 20 min of contact time using a concentration of 5 mM is 97.53% (P2) and 97.43% (P3).

#### 3.2.2. Reusability Study of P1, P2, and P3 Materials

**Figure 7:**
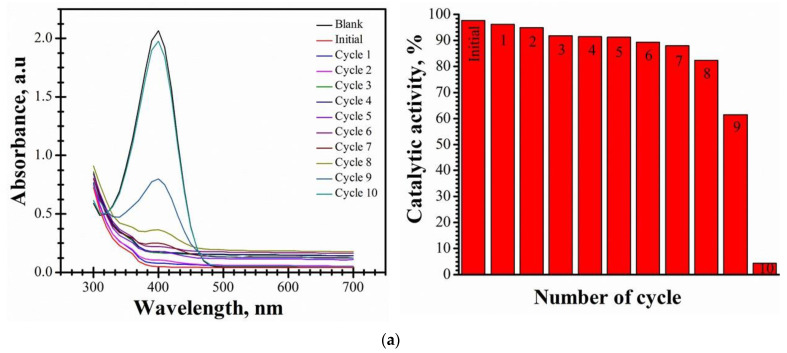

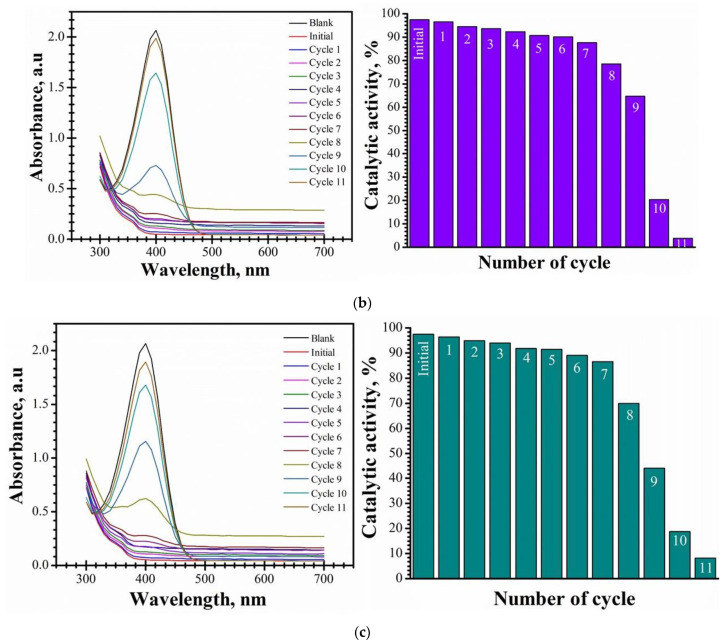
(**a**) Catalytic activity of P1 material at the 4-NP concentration of 5 mM for 10 cycles. (**b**) Catalytic activity of P2 material at the 4-NP concentration of 5 mM for 11 cycles. (**c**) Catalytic activity of P3 material at the 4-NP concentration of 5 mM for 11 cycles.

The ability of P1, P2, and P3 materials to be reused in this type of application is very important. In the case of P1 material, the reusability study was performed for 10 cycles (Figure 7a). In order to establish the reusability of P2 and P3 materials, the catalytic experiments have been repeated for 11 cycles after completing the initial cycle. The results are illustrated in Figure 7b for P2 material and in Figure 7c for the P3 material, respectively.

An important observation is that, after four cycles, all three synthesized materials show similar levels of catalytic activity for the reduction of 4-NP to 4-AP: 91.38% (P1), 92.3% (P2), and 91.72% (P3). So, no significant loss is observed after the four cycles. The catalytic activity after Cycle 6 is slightly reduced in comparison with the catalytic activity after Cycle 4: 89.3% (P1), 90.22% (P2), and 89.06% (P3). Therefore, the results obtained prove that materials can be reused for six cycles without the catalytic activity being visibly affected. After Cycle 7, the catalytic activity is 88% for P1, 87.7% for P2, and 86.49% for P3. Cycle 8 leads to a catalytic activity of 82.33% (P1), 78.51% (P2), and approx. 70% (P3). After 10 cycles, the catalytic activity of P1 material is reduced (4.31%). P2 and P3 materials show a catalytic activity of 20.47% and 18.68%, respectively. After 11 cycles, the catalytic activity of P2 and P3 materials is 3.87% and 8.27%, respectively.

The decrease in the catalytic activity of materials is due to the block of the Pt active sites to the hydrogen molecules by the adsorbed nitrophenol molecules. This result is in agreement with that obtained by Zhang and co-workers [32].

In conclusion, the P1-P3 materials have the potential to be involved in applications for the reduction of 4-NP to 4-AP, having a good catalytic activity for six cycles (approx. 90%).

Different types of materials have been investigated for the reduction of 4-NP to 4-AP. Further, a comparison of the catalytic activity of P1, P2, and P3 materials with some materials proposed in the literature for the reduction of 4-NP to 4-AP was performed. The data are summarized in Table 2 (the results regarding the catalytic activity of mentioned materials correspond to six cycles of reusability). 

By comparison, according to the data presented in Table 2, it can be highlighted that Co_x_Pt_100−x_ alloys in the shape of thin films synthesized in the present work show promising results for 4-NP reduction for more than six cycles of reusability. Additionally, the tests showed that P1, P2, and P3 materials can be involved for a higher number of reusability cycles. After eight cycles, the materials still have good catalytic activity, as we have already mentioned above: 82.33% (P1), 78.51% (P2), and approx. 70% (P3).

Ehsani and co-workers [37] pointed out that the high catalytic activity and the possibility to reuse the material as much as possible are important parameters that should be considered when we want to use a material/catalyst for any reaction. Therefore, the reduction of 4-NP to 4-AP using P1 (Co_5_Pt_95_), P2 (Co_10_Pt_90_), and P3 (Co_28_Pt_72_) materials is demonstrated in the present work.

**Figure 8:**
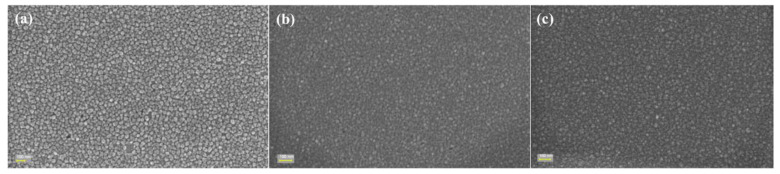
(**a**) SEM images of P1 material after 10 cycles; (**b**) P2 material after 11 cycles; (**c**) P3 material after 11 cycles.

SEM analysis, after reusability investigation, was performed to investigate the structure of the materials after reusability (Figure 8). As can be observed, the images do not reveal any changes in the structure of materials after 10 and 11 cycles, respectively.

## 4. Conclusions

In conclusion, Co_x_Pt_100−x_ (x = 5, 10, 28, and 40) alloys in the shape of thin films were successfully prepared using electrodeposition from a stable hexachloroplatinate aqueous solution. The electrodeposition was carried out at room temperature at different applied potentials. The alloy chemical composition is a function of the value of the applied potential during the synthesis, the platinum content of the material decreasing as the value of the applied potential increases. The chemical composition of the materials influences the crystallite orientation but not the materials’ crystalline structure. 

The data obtained during the study reveal that, depending on the applied electrodeposition potential, the catalytic activity levels of the Co_x_Pt_100−x_ alloys towards 4-NP reduction are different.

At 10 mM concentration, P1 material shows similar catalytic activity after 30 and 60 min of contact time. For P2, P3, and P4 materials, the catalytic activity is improved after 60 min of contact time.

The experimental results show that the process concerning the catalytic activity of P1-P3 materials is very fast when a concentration of 5 mM is used compared to a concentration of 10 mM. It requires approx. 60 min to complete the reduction of 4-NP to 4-AP.

According to the reusability results, all three materials have the potential to be used in the reduction of 4-NP to 4-AP, the materials presenting a high catalytic activity for six cycles (about 90%).

Overall, the findings of the present study show that Co_x_Pt_100−x_ synthesized materials may be employed for the reduction of phenolic compounds. However, taking into consideration the scarcity of the Pt, combined with the fact that the P3 material shows very good catalytic activity together with a low Pt content, this material can be recommended for wastewater treatment.

Future studies will concentrate on investigating the ability of Co_x_Pt_100−x_ alloys to treat wastewaters contaminated with other types of dangerous pollutants.

## Figures and Tables

**Table 1:** The synthesis conditions of materials (samples P1–P4).

Material	Electrodeposition Potential	Alloy Composition	
		% Co	% Pt
P1	−0.6 V	5	95
P2	−0.7 V	10	90
P3	−0.8 V	28	72
P4	−0.9 V	40	60

**Table 2:** A comparison of the catalytic activity between as-synthesized Co_x_Pt_100−x_ alloys and different materials from literature.

Material	Catalytic Activity, %	Reference
Palladium nanoparticles supported on maleic anhydride-acylated chitosan (Pd/MAAC)	~91	[33]
Co_3_O_4_ nanosheets	88.84	[34]
PdNi bimetallic nanoparticles (NPs) supported on graphite oxide (PdNi/GO)	74.8	[35]
Monometallic Pd/GO	59.8	
FC_900_ nanosheets	~70	[36]
P1 (Co_5_Pt_95_) thin film	89.3	This work
P2 (Co_10_Pt_90_) thin film	90.22	This work
P3 (Co_28_Pt_72_) thin film	89.06	This work

## Data Availability

Data are available upon request from the corresponding authors.
